# NEWS - REGIONS

**DOI:** 10.7189/jogh.05.020202

**Published:** 2015-12

## THE BILL AND MELINDA GATES FOUNDATION

➢ Each year, 6.3 million children die before their 5^th^ birthday – a 50% reduction compared to 1990. The BMGF aims to reduce this by a further 50%, with a US$ 776 million investment in child nutrition. According to the BMGF, under–nutrition is a major factor in half of child deaths, and it will invest in prevention strategies. These will include providing seeds and technical expertise to harvest fortified crops, agricultural techniques which conserve soil and water, training in agricultural techniques and expanding internet access to share information on crop prices and market conditions. Extra efforts will be made to reach women farmers – 50% of African farmers – who are 90% more likely to re–invest additional income in the family compared to men. The BMGF will also support the education of young women and adolescent girls about proper diet and the importance of breastfeeding, in recognition that the first 1000 days from conception are crucial for long–term physical and mental development. The BMGF will focus on India, Ethiopia, Nigeria, Bangladesh and Burkina Faso, and there will be partial matched funding from the UK. The EU has pledged US$ 3.8 billion by 2020 to fight child malnutrition. (*Time*, 3 June 2015)

➢ Bill Gates announced plans to invest US$ 2 billion into breakthrough energy projects that will reduce greenhouse gas emissions and bypass fossil fuels. Mr Gates has already invested US$ 1 billion in early–stage energy companies that are geared to preventing climate change. In making this investment, Mr Gates argues that existing clean energy sources can only curb global warming at “astronomical” costs, so innovation is desperately needed. It will probably target early–stage clean energy projects, eg, high–altitude wind power, solar chemical power and depleted uranium as a power source. Investment in renewable energy is expected to reach US$ 8 trillion over the next 25 years, outpacing investment in fossil fuels and nuclear. “Because there’s so much uncertainty and there are so many different paths, it should be like the Manhattan Project and the Apollo Project, in the sense that the government should put in a serious amount of R&D,” says Mr Gates. (*International Business Times*, 25 Jun 2015)

➢ The BMGF is a part of a major investment in Editas Medicine, which has raised US$ 120 million to further develop a new technology to edit genes to treat disease. Editas Medicine, based in Cambridge MA (USA), hopes to use the tool CRISPR–Cas9 to allow scientists to hone in on faulty genes and replace with a healthy gene. Katrine Bosley, the chief executive of Editas said “they all appreciate the vast potential of this science. The heart of the conversation we had with everybody is how you translate this very exciting but young science into treatments, into therapies.” (*Business Insider*, 10 Aug 2015)

➢ The BMGF has awarded Novavax a grant of up to US$ 89 million to support the development of the RSV F Vaccine Phase 3 clinical trials in pregnant women. It will also support regulatory licensing efforts towards pre–qualification by the WHO. Novavax will make the final vaccine affordable and accessible to people in the developing world. “Respiratory syncytial virus (RSV) is the leading cause of pneumonia in infants, and currently there are no affordable approaches to protecting children in the developing world. Maternal immunisation may provide protective antibodies to infants during the first few months of life, and we hope this vaccine will protect infants from this disease to help them live healthy, productive lives,” said Dr Keith Klugman, director of the BMGF’s Pneumonia Program. (*Street Insider*, 29 Sept 2015)

➢ Four years after its introduction, the MenAfriVac vaccine has led to the number of meningitis A cases approaching zero in the epidemic belt across Africa. This area experiences an epidemic of meningitis A every 10–15 years; in 1996–1996 there were more than 250 000 cases, 25 000 people died and many more suffered from permanent disabilities. Following this, African governments united to demand the development of an effective, affordable vaccine that could be rolled out across the region. There was a lack of private investment, as this strain of meningitis A affects some of the poorest places on earth. However, a partnership between PATH and the WHO led to the Meningitis Vaccine Project, which aims to develop and produce an effective vaccine. The partnership model has no permanent organisation, but is a coalition that is formed anew for each project, and is tailored to its specific needs. The project received US$ 70 million of funding from the BMGF. (*IRIN news*, 17 Nov 2015)

## THE GAVI ALLIANCE

➢ New figures from the WHO show that the triple–vaccine coverage rate amongst children for diphtheria, tetanus and pertussis was 81% in the 73 countries supported by GAVI. This is a 1% increase from 2013, and 21% increase from 2000, when GAVI was formed. In 2014, all GAVI–supported countries extended their triple vaccines to the pentavalent vaccine, which includes hepatitis B and *Haemophilus influenzae* type b. There were also significant gains in the coverage for pneumococcal and rotavirus immunisations. However, there are coverage variations between countries, and the sharp falls in Haiti, Côte d'Ivoire and Zimbabwe are particularly concerning. This highlights the importance of understanding these variations, both between countries and at sub–national/community levels. Moreover, the countries most affected by Ebola have experienced falls in coverage, and GAVI is working to help them rebuild their routine immunisation systems. (*allafrica.com*, 17 Jul 2015)

➢ A trial in Guinea shows that an experimental Ebola vaccine (rVSV–ZEBOV) seems to give total protection, according to a study published in *The Lancet.* The trial was based on a ring design – used by the smallpox eradication campaign – whereby each patient’s close contacts are vaccinated to halt onwards transmission. The vaccine appears to work well for 3 weeks – excellent for an outbreak situation, but there are questions over longer–term protection. The WHO is now considering whether to approve the vaccine for general use. This could enable the vaccine to be stockpiled for future outbreaks. However, GAVI will work with researchers and industry to develop second–generation vaccines, as rVSV–ZEBOV must be stored at –80°C and protects against a limited number of Ebola strains. The second–generation vaccines will target other strains and the closely–related Marburg virus, and do not require expensive storage. “This is illustrating that it is feasible to develop vaccines much faster than we’ve been doing,” says Adrian Hill, a vaccine scientist involved in testing other Ebola vaccines. (*Nature*, 31 Jul 2015)

➢ Dr Ngozi Okonjo–Iweala, Nigeria’s former Minister of Finance was appointed the new Chair Elect of GAVI. Dr Okonjo–Iweala led negotiations with the Paris Club of Creditors in 2005 that led to the clearing of US$ 30 billion of Nigeria’s external debt. She is also a former Managing Director of the World Bank, with special oversight of for Eastern Europe, Central and South Asia, and Africa. Her accolades include *Time* magazine’s European Hero of the Year (2004), and being listed as one of the 100 most powerful women in the world by *Forbes*. “I am excited to be joining GAVI during this crucial time. GAVI has a well–earned reputation as one of the leading players in global health, providing services that underpin human and economic development,” she says. (*venturesafrica.com*, 21 Sept 2015)

➢ The Republic of Korea has signed an agreement with GAVI to improve its support for childhood vaccinations in some of the world’s poorest countries. The Republic of Korea will provide an additional US$ 3 million between 2015 and 2017. These funds will be used to finance immunisation programmes in 73 countries, designed to protect children against diarrhoea and pneumonia. Mr Lee Yongsoo, of the Republic’s Ministry of Foreign Affairs said “health and children are a priority or Korea’s development co–operation policy and GAVI is our partner. We will continue to strengthen our co–operation with GAVI in the years to come.” (*Vaccine News*, 7 Oct 2015)

➢ Data from the WHO shows a 79% reduction in measles deaths, from 546 800 in 2000 to 114 900 in 2014, with an estimated 17.1 million lives being saved. The reduction is mainly due to expanded vaccine coverage, and is one of the main contributors to reducing child mortality and progress towards MDG4. However, coverage rates have stagnated at 85% between 2010 and 2014 (despite large increases up to 2010) so the 2015 global milestones and WHO measles elimination goal will not be achieved on time. In 2014, mass vaccination campaigns led by governments with support from the Measles and Rubella Initiative and GAVI, reached 221 million children, and since 2000 these campaigns have enabled 2 billion children to receive a supplementary dose of measles vaccine. “Despite the welcome reduction in measles deaths, this highly infectious disease continues to take a terrible toll on the lives of children around the world. A co–ordinated approach that puts stronger routine immunisation at its core will be central to getting measles under control and securing further reductions in mortality from this vaccine–preventable disease,” says Dr Seth Berkley, CEO of GAVI. (*Business Standard*, 13 Nov 2015)

## THE WORLD BANK

➢ According to Subhash Chandra Garg, the World Bank Executive Director for India, Bangladesh, Bhutan and Sri Lanka, India could become a multi–trillion dollar economy with a per–capita income of US$ 40 000 (compared to its current US$ 2000) by 2050. This will require economic growth of at least 7% a year over the next 30–35 years, the transformation of India’s agricultural sectors, and boosting its tourism, manufacturing, services and health care. People must move out of agriculture into services and manufacturing, whilst ensuring that agricultural production increases. India must exploit its demographic dividend of a large young population, and invest in training these workers to provide exportable skills. Mr Garg noted how the World Bank is partnering with the Indian government through initiatives such as Smart Cities and the Swachh Bharat campaign to realise the vision of a strong and prosperous nation. (*Economic Times*, 20 Jul 2015)

➢ The World Bank approved a US$ 700 million investment in an offshore gas project in Ghana to address its electricity shortages, which frequently cause power blackouts. This consists of a US$ 500 million guarantee to support regular gas purchases, and US$ 200 million to help secure private financing. Gas production is due to start in 2018, with a capacity of 1000MW, and enables Ghana to switch its energy mix from oil to natural gas. This will reduce its yearly oil imports by up to 12 million barrels, and cut yearly CO_2_ emissions by 1.6 million tonnes. The IMF had described Ghana’s electricity crisis as “the single most important risk” to its economic development. In welcoming the investment, Ghana’s finance minister Seth Terkper said “this project is an essential element of the driver towards consolidating our middle–income status, and will help secure our natural gas resources for a more affordable and reliable power supply.” (*Business Green*, 30 Jul 2015)

➢ The World Bank Report *Going universal: how 24 developing countries are implementing universal health coverage reforms from the bottom up* shows how universal health coverage (UHC) programmes are reducing the number of people impoverished by health care costs. These programmes, from countries such as Ethiopia, Ghana, Peru, Jamaica and Vietnam, cover more than 2 billion people, and were found to be “new, massive and transformational”, although greater investment is required. Both the WHO and World Bank recommend that countries implementing UHC should provide 80% of the population with essential health services, and that everyone should be shielded from impoverishing health payments. Tim Evans, senior director of health, nutrition and population at the World Bank Group, said UHC was a triple win. “It improves people’s health, reduces poverty and fuels economic growth,” he said. (*Public Finance International*, 25 Sept 2015)

➢ A new report from the World Bank shows that the number of people worldwide living in extreme poverty will fall to 9.6%–or 702 million people–of the world’s population in 2015 – the first time it has ever fallen below 10%. In 1999, 29% of all people lived in extreme poverty, falling to 13% by 2012. According to Mr Kim, the World Bank president, the fall is due to strong economic growth in developing countries, coupled with investments in health, education and social safety nets. Extreme poverty was previously defined as living on US$ 1.25/d, but the World Bank has revised this to US$ 1.90/d to take account of inflation. This reduction increases the momentum towards ending extreme poverty. However, the World Bank is cautious over the obstacles faced, including the growing concentration of poverty in sub–Saharan Africa and reducing extreme poverty when economic growth falters in emerging economies, although it welcomes the sharp falls in Asia and South America. The development agency, Oxfam, highlighted that 702 million people living in extreme poverty is unacceptably high, and that much needs to be done. (*ABC news*, 5 Oct 2015)

➢ In its report *Shock Waves: Managing the Impact of Climate Change on Poverty*, the World Bank highlights how climate change could force more than 100 million people beneath the poverty line by 2030, threatening the goal of eliminating extreme poverty. The world’s poorest people are more exposed to climate–related disasters (eg, heat waves, flooding and drought), and can lose resources when dealing with these disasters. It is estimated that climate change could increase Africa’s food prices by up to 12% by 2030, and 70% in 2080 – devastating for poor households where food is 60% of total expenditure. It calls for money saved on eliminating fossil fuels to be invested in assistance programmes, and warns that without immediate adoption of adaptation, mitigation and emission–reduction policies, rising temperatures and greenhouse gases will devastate vulnerable populations. (*Tech Times*, 27 Nov 2015)

## UNITED NATIONS (UN)

➢ According to the UN Millennium Development Goals (MDG) report, the MDGs have lifted 1 billion people out of poverty since 2015, making it one of the most successful anti–poverty movements in history. Sub–Saharan Africa is lagging behind, with most citizens facing several inequality although there has been marked improvements in some areas eg, child mortality. The report urges the continent to accelerate progress, supported by improved tax and revenue collection to fund projects. Overall, the numbers living in extreme poverty fell from 1.9 billion in 1990 to 836 million in 2015. Gender parity has been achieved in many areas, especially in schooling and new HIV infections fell 40% from 3.5 million to 2.1 million. The MDGs will be replaced by new Sustainable Development Goals later in 2015. (*Voice of America*, 9 Jul 2015)

➢ According to the UN Electoral Observation Mission, Burundi’s infrastructure is not conducive for holding creditable elections. Despite two postponements, the incumbent President Pierre Nkurunziza won a third term. In a statement, MENUB said that the country’s Constitutional Court ruling on the President’s availability to stand for a third term did not resolve presidential term limits, but instead worsened tensions. There was no balanced media coverage for all political parties, and freedom of expression, debate and assembly were curtailed. Following the elections, 100 people have died in protests and 170 000 people have fled the country. (*Sahara Reporters*, 28 Jul 2015)

➢ The UNHCR stated that nearly 300 000 refugees and migrants have crossed the Mediterranean Sea to Europe in 2015. People are arriving in groups of 300–400, and up to 3000 asylum–seekers arrive at the Macedonian border each day. Most people are fleeing violence in Syria, Afghanistan and Iraq. People are often exhausted and traumatised on arrival, and need humanitarian assistance. Most refugees head for Germany and Sweden, which accepted 43% of asylum–seekers. Germany expects to receive 800 000 refugees in 2015. A UNHCR spokesperson, Melissa Fleming, says that this is not sustainable and that a more decent and humanitarian distribution of these people amongst Europe’s 28 member nations is needed. The UNHCR also calls for improved legal avenues, eg, more student and work visas, and resettlement opportunities, to lessen the number of people undertaking these dangerous journeys. (*Voice of America*, 25 Aug 2015)

➢ According to the UN, the Taliban’s insurgency is at its widest since 2001, earlier rating the threat level in half of Afghanistan’s districts as either “high” or “extreme”. The Taliban has inflicted ambushes and roadblocks on Highway One, a ring–road connecting Afghanistan’s major cities, and have repeatedly cut the highway in government strongholds. In many areas that are technically under government control, government forces hold only the government buildings and are under attack from insurgents. The UN data suggests increased Taliban activity in areas with hitherto limited presence. The UN has evacuated some staff from Afghanistan, and other aid agencies are following this. (*New York Times*, 11 Oct 2015)

➢ Ban Ki–Moon, the UN Secretary–General, congratulated Aung Suu Kyi over the National League for Democracy’s (NLD) victory in Myanmar’s elections. Mrs Suu Kyi is the NLD chairperson, and the NLD won over 50% of the seats in the Union Parliament. Ban said that the UN would support Myanmar’s democratic reform, and hailed the elections as a “defining moment in the reform process, and have opened up real potential for Myanmar to thrive as an inclusive, harmonious, multi–ethic and multi–religious democracy.” (*Xinhua*, Nov 2015)

## UN AIDS AND THE GLOBAL FUND

➢ According to UNAIDS, the goal of 15 million people on HIV treatment by the end of 2015 was reached in March–9 months ahead of schedule. This compares to fewer than 700 000 people in 2000. The global response has prevented 30 million new infections and 8 million deaths since 2000. New infections fell from 2.6 million per year to 1.8 million, and AIDS–related deaths fell from 1.6 million to 1.2 million over the same period. Global HIV investment has risen from US$ 4.8 billion to US$ 20 billion. Despite this, progress has been slower in some areas, with HIV status awareness being a major barrier to treatment and treatment for HIV–positive children lags behind adults – although the gap is narrowing. Ban Ki–moon, the UN Secretary–General, says “the world has delivered on halting and reversing the AIDS epidemic. Now we must commit to ending the AIDS epidemic as part of the Sustainable Development Goals.” UNAIDS recommends front–loading investment to “sprint” towards the target of ending the AIDS epidemic by 2030. (*BBC*, 14 Jul 2015)

➢ A statement by the UNAIDS Executive Director, Mr Michel Sidibé, announced a 3.5% reduction in the cost of HIV early diagnostic tests for children, bringing them to US$ 9.40. This reduction, in partnership with Roche Diagnostics, will help scale–up diagnostic and treatment services for HIV–positive children, in line with the 90–90–90 target [ie, by 2020, 90% of all HIV–positive people will know their status; 90% of all HIV–positive people will receive sustained antiretroviral therapy; and 90% of all people receiving antiretroviral therapy will have viral suppression]. The WHO recommends that all children exposed to HIV receive screening within the first two months of life, but currently screening only reaches 50% of these children due to financial constraints. This had led to a major gap in HIV treatment access, with 32% of HIV–positive children receiving antiretroviral treatment, compared to 41% of adults. Without treatment, 50% of HIV–positive children would die by the age of 2 years, and the majority of the remaining 50% would die by the age of 5 years. (*allafrica.com*, 20 Jul 2015)

➢ Dr Adesina Fagbenro, the Southwest Co–ordinator for the UK’s Department for International Development (DfID) warned that Nigeria is at risk of losing access to the US$ 400 billion Global Fund. The warning was prompted by Dr Fagbenro’s concern that the government is failing to understand the technical issues around accessing the fund. He stated that it is difficult to access data from ministries and other agencies in many states–and the lack of data evidence will hamper the presentation of a planning proposal. “We have to track the indicators and measure our performance on such issues. Lagos is now enjoying the status of being able to receive budget support. It is only when progress is noticed that international financing agencies will come around to support you. If you don’t get it right, you cannot get the needed support,” said Dr Fagbenro. (*The Nation*, 25 Aug 2015)

➢ At the Oct 2015 G7 summit, health ministers from the G7 group of most developed countries agreed to the Berlin Declaration on Antimicrobial Resistance, which aims to support LMICs to develop national antimicrobial resistance plans. This requires a three–fold approach: improving infection prevention; protecting the effectiveness of existing and future antimicrobials; and researching new antimicrobials, vaccines, treatment alternatives and rapid diagnostic tools. The ministers also committed to learning lessons from Ebola, and reiterated their support for the WHO International Health Regulations. They have offered to work with 60 countries to implement IHR. The G7 ministers will work closely with organisations such as the Global Fund, GAVI and WHO on this initiative. (*Intellectual Property Watch*, 12 Oct 2015)

➢ At the opening session of the 19th European AIDS conference, Prof Kazatchkine (the UN Special Envoy on HIV/AIDS in Eastern Europe and Central Asia) called for Europe to increase HIV prevention and treatment activities to meet the UNAIDS target of 90% of those diagnosed with HIV to receive treatment, and 90% of those on treatment to have fully suppressed viral loads by 2020. This would result in 73% of HIV–positive people having undetectable viral load–if this target is achieved by 2020 it would end the AIDS epidemic by 2030. Eastern Europe, Central Europe and Western Europe have different experiences of the HIV epidemic. In Eastern Europe, HIV is more prevalent amongst people who inject drugs, although heterosexual transmission is increasing – and prevention and harm reduction services are limited. HIV is concentrated amongst gay men and injecting drug users in central Europe, with limited services provided to those groups. And Western Europe has a stable infection rate, despite strong health care and support. Prof Kazatchkine calls for intensified efforts and more resources to tackle HIV, and to understand and address weaknesses in the current response. (*aidsmap.com*, 22 Oct 2015)

## UNICEF

➢ UNICEF’s report *2014 State of Children in Pakistan* shows that 7.1% of Pakistan’s children die before their 1st birthday, and 9.1% of children die before their 5th birthday. Although these statistics are a massive improvement on 1990–when 10.6% of children died before their 1st birthday, and 13.8% died before their 5th birthday–they are much worse than comparable countries eg, India. The report states that a lack of clean drinking water and sanitation are two of the biggest hurdles in reducing child mortality. Children growing up in poverty are also less likely to access basic services, or to benefit from preventative initiatives or protection mechanisms. (*Express Tribune*, 9 Jul 2015)

➢ According to UNICEF, more than 70 000 births in Liberia went unrecorded due to the Ebola crisis–a 40% reduction compared to 2013. The fall in registrations is caused by the closure of maternity wards as health workers were infected with the virus. However, births may also have fallen during the outbreak which could be a contributing factor. UNICEF is working with the Liberian government to register all babies unrecorded during the epidemic, and to help rebuild the country’s shattered health system. “Children who have not been registered at birth officially don’t exist. Without citizenship, children in Liberia, who have already experienced terrible suffering because of Ebola, risk marginalisation because they may be unable to access basic health and social services, obtain identity documents, and will be in danger of being trafficked or illegally adopted,” says Sheldon Yett, UNICEF’s representative in Liberia. (*Yahoo news*, 31 Jul 2015)

➢ In an open letter published in the *Huffington Post*, Anthony Lake, Executive Director of UNICEF, addressed a girl born into poverty during September 2015. This is the month when world leaders meet about the Sustainable Development Goals. These goals should give this girl–and the other 500 million children living in extreme poverty – the same right to a fair chance in life, and to close the gap between their prospects and those who are better off. He called on these children to hold world leaders accountable for any failures to meet these goals. He also addressed a boy not born into poverty during September 2015, to remember that these goals are universal goals and to also hold world leaders accountable for any failure. (*Huffington Post*, 1 Sept 2015)

➢ A joint release from UNICEF, the World Food Program and the Food and Agriculture Programme calls for unrestricted humanitarian access to prevent famine in parts of Unity State in South Sudan. The 2–year civil war has left nearly 4 million people at risk of starvation, and aid agencies are struggling to reach these people. Fighting in South Sudan has displaced more than 2 million people, and livelihoods have been devastated by high inflation rates, market disruption, displacement, loss of livestock and agricultural production. Food shortages are exacerbated by pending harvest shortfalls in Uganda, the Sudan and Ethiopia, which will further increase food costs in South Sudan. (*Humanosphere*, 2 Nov 2015)

➢ More than 500 000 children face life–threatening malnutrition in Yemen according to UNICEF. The UN has designated Yemen as a high–level humanitarian crisis, with more than 80% of its people on the brink of famine and 10 million children are in need of urgent relief–and aid deliveries are being severely restricted. There is no end in sight to the conflict, and the UN–backed peace talks between the government and the Houthi rebels have no set date for beginning negotiations. In the meantime, 2 million Yemeni children cannot attend school, and Yemen’s malnourished children risk stunting with the resultant risk of lifelong impaired cognitive functioning. “The situation continues to worsen. What we need is a political settlement urgently,” says Mr Anthony Lake, UNICEF’s executive director. (*Middle East Eye*, 17 Nov 2015)

## WORLD HEALTH ORGANIZATION (WHO)

➢ According to a WHO report, only 33 countries have raised tobacco taxes to a minimum of 75% of retail price; none among these are low– and middle–income countries (LMICs). Increasing tobacco taxes is a proven method to reduce demand and deaths from tobacco use. It could provide an innovative model for tackling the growing burden of non–communicable diseases (NCDs), which receive 1.7% of global health expenditure. On current trends, NCD funding will not reach the level required to tackle NCDs in LMICs–estimated by the WHO to be US$ 11.4 billion. Whilst many countries channel existing tobacco revenues into health initiatives, there is a tendency for countries to focus on specific diseases, eg, HIV/AIDS. As LMICs struggle with tax collection, this underlines the need to boost their tax collection and administration capacities. (*Devex*, 8 Jul 2015)

➢ According to a WHO update, no new cases of Ebola were reported in Liberia (where it had resurfaced after being declared free of Ebola in May 2015) or in recent hotspots in West Africa, where no new cases had been reported for several days. Half of the confirmed cases were from the capitals of Guinea and Sierra Leone. Contact tracing shows that almost all new cases have arisen from registered contacts of previous cases. The cases which had earlier resurfaced in Liberia were most likely caused by the re–emergence of the virus in surviving patients. The WHO noted that two new health worker infections were reported in Guinea and one from Sierra Leone. (*UN*, 22 Jul 2015)

➢ The WHO is expected to recommend that all HIV–positive people should receive antiretroviral (ART) drug treatment immediately upon diagnosis. This would raise the number of people eligible for treatment to 37 million from 28 million; only 15 million people currently receive treatment. This recommendation is prompted by research which shows that starting treatment when the immune system is strong reduces the risk of death or serious health problems by 57%, and reduces the risk of transmission by 93%. The WHO is also expected to recommend ART for uninfected people at higher risk of infection, eg, sex workers. Although it is simpler to treat all HIV–positive patients rather than basing treatment on CD4 cell counts, there are challenges with managing treatment compliance at the early stage of infection, with the ensuing risk of drug resistance; the biggest challenge is ensuring that people who are infected receive treatment, as only 50% of infected people know their status. (*scidev.net*, 28 Jul 2015)

➢ According to the WHO, two children have been paralysed in the first polio outbreak in Europe for five years. Both cases were in the Ukraine, where only 50% of children are fully immunised. The risk of the virus spreading is “high”, and the WHO calls for the outbreak to be rapidly controlled. It is likely that other children have been infected without developing symptoms. The outbreak arose from the weakened strain of polio virus, which can mutate if immunisation levels are too low. The WHO recommends that everyone visiting the region is fully vaccinated, and that all residents and anyone staying for more than one month receives a polio booster. (*BBC*, 2 Sept 2015)

➢ According to Médecins Sans Frontières (MSF), 5 million people are bitten by snakes each year, and 10% will die or suffer permanent disability. Snake bite antidotes saves thousands of people each year, but the main antidote–Fav–Afrique– is due to run out and will not be replaced in the short–term. Its manufacturer, Sanofi, stopped production in 2014 and the last batch will expire in June 2016. The technology may be transferred to another company, but this will not be completed until late 2016 and the product will not be available until late 2018. Despite the high fatality rates, snake venom antidotes have not been regarded as a priority by funders. MSF blame this situation on low awareness and lack of prioritization, and highlight that the WHO does not have a specialist in this area. (*Medical News Today*, 8 Sept 2015)

